# Long noncoding RNA ZEB1-AS1 epigenetically regulates the expressions of ZEB1 and downstream molecules in prostate cancer

**DOI:** 10.1186/s12943-017-0711-y

**Published:** 2017-08-23

**Authors:** Wenjing Su, Miao Xu, Xueqin Chen, Ni Chen, Jing Gong, Ling Nie, Ling Li, Xinglan Li, Mengni Zhang, Qiao Zhou

**Affiliations:** 1 Department of Pathology and Laboratory of Pathology, State Key Laboratory of Biotherapy, West China Hospital, West China Medical School, Sichuan University, 37 GuoXueXiang, Chengdu, 610041 China; 2 Department of Pathology, Shandong Provincial Hospital affiliated to Shandong University, 324 Jingwu Road, Jinan, 250021 China

**Keywords:** ZEB1-AS1, ZEB1, miR200c, BMI1, Prostate cancer, Long non-coding RNA

## Abstract

**Background:**

Emerging studies show that long noncoding RNAs (lncRNAs) play important roles in carcinogenesis and cancer progression. The lncRNA ZEB1 antisense 1 (ZEB1-AS1) derives from the promoter region of ZEB1 and we still know little about its expressions, roles and mechanisms.

**Methods:**

RACE was used to obtain the sequence of ZEB1-AS1. RNA interference was used to decrease ZEB1-AS1 expression. Adenovirus expression vector was used to increase ZEB1-AS1 expression. CHIP and RIP were used to detect the epigenetic mechanisms by which ZEB1-AS1 regulated ZEB1. CCK8 assay, wound healing assay and transwell assay were used to measure proliferation and migration of prostate cancer cells.

**Results:**

In this study, in prostate cancer cells, we found that RNAi-mediated downregulation of ZEB1-AS1 induced significant ZEB1 inhibition while artificial overexpression of ZEB1-AS1 rescued ZEB1 expression, which means that ZEB1-AS1 promotes ZEB1 expression. Also, ZEB1-AS1 indirectly inhibited miR200c, the well-known target of ZEB1, and upregulated miR200c’s target BMI1. Mechanistically, ZEB1-AS1 bound and recruited histone methyltransferase MLL1 to the promoter region of ZEB1, induced H3K4me3 modification therein, and activated ZEB1 transcription. Biologically, ZEB1-AS1 promoted proliferation and migration of prostate cancer cells.

**Conclusions:**

Collectively, ZEB1-AS1 functions as an oncogene in prostate cancer via epigenetically activating ZEB1 and indirectly regulating downstream molecules of ZEB1.

## Background

The zinc finger E-box-binding protein 1 (ZEB1, also known as ZFHX1A, AREB6, or ΔEF1) is a transcriptional factor that plays important roles in physiology and tumorigenesis. Overexpression of ZEB1 is associated with the malignant behaviors of tumors such as endometrial carcinoma, bladder cancer, colorectal cancer and also prostate cancer [1–4]. As a famous epithelial-mesenchymal transition (EMT) promoter, ZEB1 not only participates in tumor biological behaviors such as cell apoptosis, chemoresistance, invasion and metastasis, but also induces stem-cell properties [5–9].

B cell-specific Moloney murine leukemia virus insertion site 1 (BMI1), a member of the polycomb repressive complex 1 (PRC1), is a crucial regulator in prostate stem cell self-renewal [10]*.* BMI1 is over-expressed in prostate cancer with adverse pathologic and clinical features and the presence of BMI1 in prostate cancer specimens often indicates metastatic disease and a high probability of unfavorable therapeutic outcome [11, 12]*.* Also because BMI1 is directly repressed by miR200c [13], the transcription of which is directly inhibited by ZEB1, we speculated that the ZEB1-miR200c-BMI regulation pathway may play an important role in prostate cancer development and progression.

Long noncoding RNAs (lncRNAs) are functional noncoding RNAs that are transcribed throughout eukaryotic genomes, the lengths of which are usually greater than 200 nt. Previous studies showed that antisense transcripts could regulate the transcription and translation of their sense strand genes. For instance, Xist, a lncRNA derived from the inactive X, plays an essential role in X inactivation in mammals, and is itself controlled by a complex interplay of other lncRNAs such as Tsix, which is transcribed from the antisense strand of Xist [14, 15].

The antisense long non-coding RNA ZEB1-AS1 gene, which is located in physical contiguity with ZEB1, positively regulates the expression of ZEB1, promotes tumor progression and predicts poor prognosis in hepatocellular carcinoma [16]. However, the functions and mechanisms of ZEB1-AS1 in other malignancies are still largely unknown. We thus speculated the effects of ZEB1-AS1 on ZEB1 and ZEB1 downstream molecules in prostate cancer cells and detected the mechanisms.

## Methods

### Cell lines and cell culture

PC3, DU145, NCI-H1299 and A549 were maintained in RPMI1640 with 10% FBS (Life Technologies, Carlsbad, CA). T98G, U251, A375, A875,HUVEC, HBE, SK-MES-1, HeLa, 293 and LO2 were maintained in DMEM with 10% FBS.

### Clinical specimens

Prostate cancer specimens and the corresponding adjacent noncancerous tissues were obtained from Shandong Provincial Hospital affiliated to Shandong University with informed consent. The protocols used in the study were approved by the Medical Ethics Committee, Shandong Provincial Hospital Affiliated to Shandong University.

### Rapid amplification of cDNA ends (RACE)

5′ RACE and 3′ RACE were performed according to routine protocols [17]. In 3′ RACE, cDNAs were generated using an Oligo-dT primer (T20) that complemented the natural polyA tail of mRNAs. PCR was then used to amplify cDNA product from the 3′end of ZEB1-AS1 with a sense specific primer and T20. In 5′ RACE, an antisense gene specific primer was used to produce cDNA from the 5′ end of ZEB1-AS1. Next, a string of identical nucleotides (dATP) were added to the 3′ end of the cDNA. PCR was then carried out to amplify cDNA from the 5′ end using an antisense specific primer and T20. We used a priming strategy in which both the 5′ and 3′ RACE reactions were primed using the same primer sequence, albeit reverse complemented, to ensure amplification of a contiguous long transcript.

### RNA interference

siRNA vectors of ZEB1-AS1 and ZEB1 were constructed as previously described [18] with the following siRNA sequences: ZEB1-AS1-siRNA1 (GGACCAACTTTATGGAATA), ZEB1-AS1-siRNA2 (GCTGAAGTCTGATGATTTA), ZEB1-siRNA1 (TGATCAGCCTCAATCTGCA) and ZEB1-siRNA2 (GCTGAGAAGCCTGAGTCCT). Corresponding control siRNA plasmids were prepared with scrambled sequences: CON-siRNA1 (GACCTACAACTACCTATCA) and CON-siRNA2 (GTGGACACCCGATAAGTTT). Cells with stable transfection of ZEB1-AS1-siRNA1, ZEB1-AS1-siRNA2, ZEB1-siRNA1, ZEB1-siRNA2, CON-siRNA1 and CON-siRNA2 plasmids (designated as ZEBAS1-KD1, ZEBAS1-KD2, ZEB1-KD1, ZEB1-KD2, RNAi-CON1 and RNAi-CON2, respectively) were selected with G418 as described [18]. The si-MLL1 sequence was as follows: 5′-GGACAAGAGTAGAGAGAGA-3′.

### Recombinant adenoviral vectors for overexpression of ZEB1-AS1 and miR200c

The recombinant adenoviral vectors were constructed as previously described [19] and named as AD-ZEBAS1 and AD-miR200c, respectively.

### Stem-loop reverse transcription, conventional reverse transcription-PCR and real-time quantitative PCR

Stem-loop RT-PCR for mature miR200c, conventional RT-PCR and real-time quantitative PCR were carried out as previously described [19]. The PCR primers used and the product lengths were as follows: miR200c (5′-GCATAGCCCGTAATACTGCCGGGTA-3′, 5′-GTGCAGGGTCCGAGGT-3′, 67 bp), ZEB1-AS1 (5′-TCCCTGCTAAGCTTCCTTCAGTGT-3′, 5′- GACAGTGATCACTTTCATATCC-3′, 340 bp), ZEB1 (5′-CGCAGTCTGGGTGTAATCGTAA-3′, 5′-GACTGCCTGGTGATGCTGAAA-3′, 273 bp), BMI1 (5′-ATCTGTATGCCTAAAAGCGGG-3′, 5′- GGTAAGCAAGGCTCAACATA-3′, 261 bp), GAPDH (5′-GGAGCGAGATCCCTCCAAAAT-3′, 5′- GGCTGTTGTCATACTTCTCATGG -3′, 197 bp).

### Western blot

Western blot was carried out as previously described [19]. The primary antibodies used were as follows: ZEB1 (rabbit polyclonal, 1:500, Sigma-Aldrich, Saint Louis, MO), BMI1 (rabbit polyclonal, 1:1000, Epitomics, Burlingame, CA), GAPDH (mouse monoclonal, 1:10,000, Kangcheng, Shanghai, China). Horseradish peroxidase–labeled secondary antibodies were from Zymed Laboratories (Zymed/Invitrogen, Carlsbad, CA).

### Luciferase reporter construct, site-directed mutagenesis and dual reporter gene assay

The seed sequences CAGTATT (719–725 nt of BMI1 3′-UTR) with flanking sequences were amplified from genomic DNA of PC3 cells. PCR product was cloned into pMD18-T and then subcloned into the 3′-UTR downstream of the luciferase coding sequence and designated as pGL3-BMI1-UTR. A construct with site-directed mutation of the corresponding seed sequence GTCATAT was prepared by overlapping PCR and designated as pGL3-BMI1-MUT. Dual reporter gene assay was performed as described [19]. Briefly, cells were cultured in 24-well plates and transfected with the reporter constructs. Cells were infected with AD-miR200c and AD-control 4 h after transfection, collected 24 h later, and the firefly and Renilla luciferase activities were assayed.

### Chromatin Immunoprecipitation (ChIP)

ChIP assays were performed as previously described [18] with anti-H3K4me3 antibody (rabbit monoclonal; Merck Millipore) or rabbit nonimmune IgG (as negative control). The specific primers used for the promoter fragment of ZEB1 (ZEB1-pro) were 5′-CGTAGAGCGAGAGCCTCTAGGTGTAAG-3′ and 5′-CCTCTCGCTTGTGTCTAAATGCTCGAG-3′. Double-stranded small-interfering RNAs (siRNAs) and control (si-CON) were designed, synthesized, and purified (RiboBio, Guangzhou, China).

### RNA Immunoprecipitation (RIP)

Cells were pelleted and lysed. The product was sonicated and the supernatant was treated with sheared salmon sperm DNA (Invitrogen, Carlsbad, CA) and protein A/G-Sepharose (Santa Cruz, CA). Immunoprecipitation was performed overnight at 4 °C with 5μg anti-MLL1 monoclonal antibody or the control isotype IgG (Lab Vision Corp., Fremont, CA) and then with protein A/G-Sepharose and salmon sperm DNA. Precipitates were washed, and extracted with 1% SDS and 0.1 M NaHCO_3_. Eluates were pooled and heated. RNA fragments were purified, digested with DNase and used as template for RT-PCR. The primers for ZEB1-AS1 were 5′-TCCCTGCTAAGCTTCCTTCAGTGT-3′ and 5′- GACAGTGATCACTTTCATATCC -3′.

### CCK8 assay

100 μl cell suspensions (3000 cells/well) was dispensed in 96-well plates and pre-incubated for 24 h in an incubator (humidified atmosphere, 37 °C, 5% CO_2_). Treated or un-treated cells were incubated as appropriate. 10 μl CCK8 (Roche Diagnosis, Mannheim, Germany) was added to each well, incubated for 2 h in the incubator. The absorbance at 460 nm (A460) was examined using a scanning multi-well spectrophotometer (Bio-Tek, USA).

### Wound healing assay

Cells were seeded in 6-wells plates and when the cell reached the density of about 80–90%, an identical wound was made across the center of the well with a 200 μL plastic pipette tip. The remaining cells were washed with PBS and then cultured in medium containing 2% FBS. At 48 h after wounding, the wound was photographed with a microscope.

### Transwell assay

Cell migration ability was assessed with transwell assay, which was performed in 24-well plates with poly-carbonate transwell filters (Corning, USA). Cells were seeded in the upper part at a density of 1.0 × 10^5^ cells/ml in 0.2 mL of 1% FBS medium. After incubated for 48 h, cells on the upper surface of the well were scraped off with a cotton swab, and cells on the lower surface were fixed, stained and counted.

### Statistical analysis

The PASW18.0 program was used for statistical analysis.

## Results

### Identification and characterization of ZEB1-AS1

**Fig. 1:**
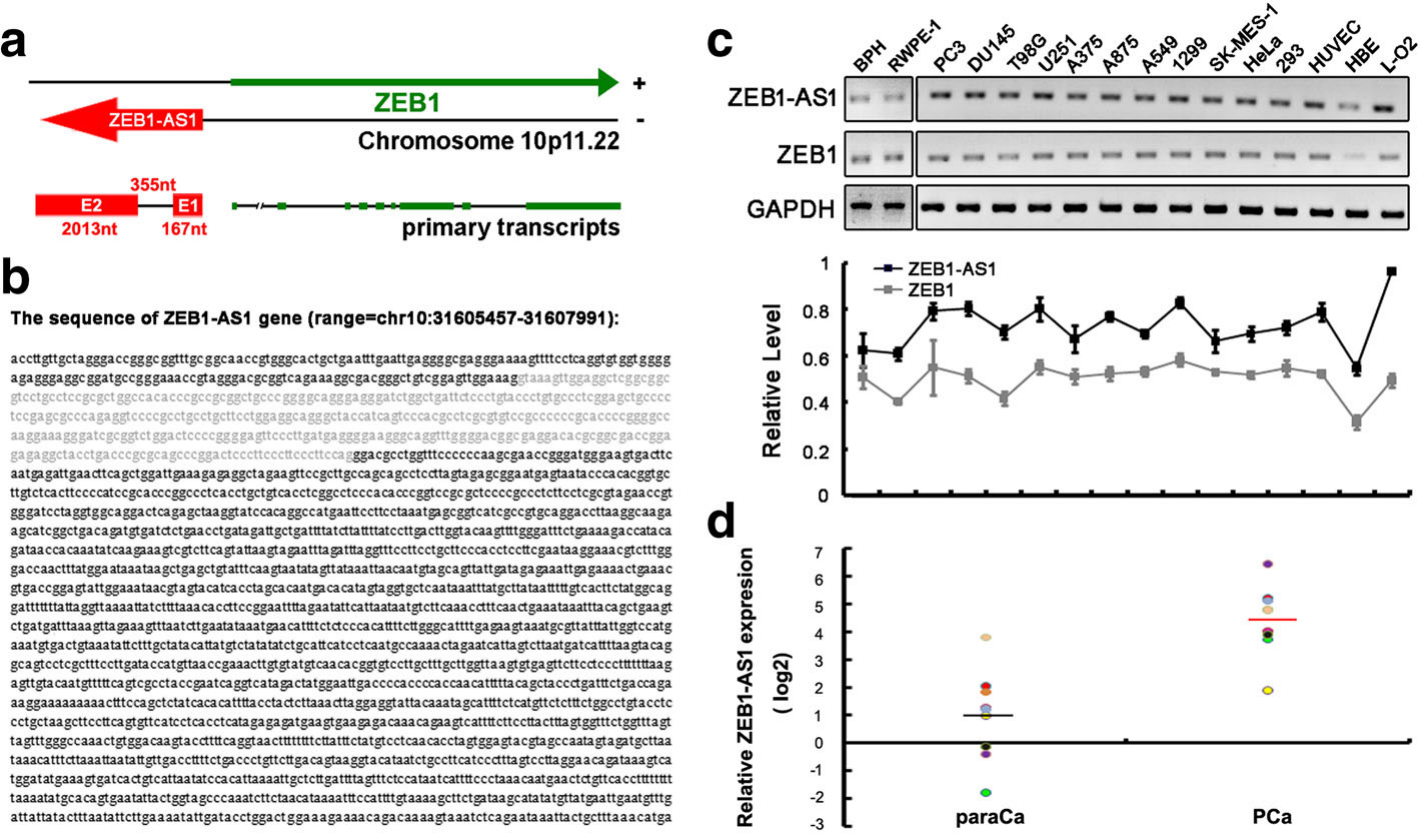
Gene structures and expressions of ZEB1-AS1 and ZEB1. **a**, schematic diagram of ZEB1-AS1 and ZEB1 structures. ZEB1-AS1 (upstream, red) was transcribed from the promoter region of ZEB1 (downstream, green), with a conversed orientation of ZEB1. The full length of ZEB1-AS1 was 2535 nt, including two exons (E1, 167 nt; E2, 2013 nt) and one intron (355 nt). **b**, the full sequence of ZEB1-AS1. Nucleotides labeled by black color were exons and those of gray were intron. **c**, RNA levels of ZEB1-AS1 and ZEB1 in a series of cell lines. RT-PCR analysis (with *GAPDH* as control) showed expression levels of ZEB1-AS1 and ZEB1 in benign prostate hyperplasia sample BPH, normal prostate epithelial cell RWPE-1, prostate cancer (PC3, DU145), glioma (T98G, U251), melanoma (A375, A875), pulmonary cancer (A549, 1299, SK-MES-1), cervical cancer (HeLa), 293, HUVEC, liver cancer (LO2) and normal human bronchial epithelial cell (HBE) (upper). Grey value analysis showed synchronous expression levels of ZEB1-AS1 and ZEB1 (lower). **d**, RNA levels of ZEB1-AS1 in prostate cancer specimens. Q-PCR analysis was used to detect the expression of ZEB1-AS1 in 9 pairs of fresh prostate cancer specimen (PCa) and the corresponding paracancerous tissue (paraCa). There was a significant difference between the two groups

The bioinformatics database (http://genome.ucsc.edu//) was used to analyze transcriptional peaks in the promoter and coding regions of ZEB1 gene. A series of primers were used in PCR amplification to identify ZEB1-AS1, which derived from the promoter region of ZEB1 gene (−110 ~ −2646 bp). Rapid amplification of cDNA ends (RACE) was employed to determine the transcriptional start and termination sites of ZEB1-AS1. The transcription direction of ZEB1-AS1 was opposite to that of ZEB1. The full length of ZEB1-AS1 was shown to be of 2535 nt, including two exons (E1, 167 nt; E2, 2013 nt) and one intron (355 nt) between the two exons (Fig. 1a and b). Bioinformatics analysis with Coding Potential Calculator (http://cpc.cbi.pku.edu.cn/) further suggested ZEB1-AS1 as a noncoding RNA, which is in accordance with the previous studies.

The levels of ZEB1-AS1 and ZEB1 were examined by RT-PCR, which showed high levels of ZEB1-AS1 and ZEB1 in prostate cancer (PC3, DU145), glioma (T98G, U251), melanoma (A375, A875), lung cancer (A549, 1299, SK-MES-1) and cervical cancer (HeLa) cells. The 293, HUVEC and LO2 cells also showed higher levels of ZEB1-AS1 and ZEB1 than those in benign prostate hyperplasia sample BPH, normal prostate epithelial cell RWPE-1 and human normal bronchial epithelial cell HBE (Fig. 1c, upper). Positive correlation between ZEB1-AS1 and ZEB1 was shown in Fig. 1c (lower). Also, the expression of ZEB1-AS1 was detected in 9 pairs of fresh prostate cancer specimen (PCa) and the corresponding paracancerous tissue (paraCa). With Q-PCR, we detected significant higher ZEB1-AS1 level in prostate cancer specimens (*P* < 0.05, Fig. 1d). In situ hybridization also showed higher ZEB1-AS1 expression in prostate cancer specimens, but the difference was not statistically significant (data not shown).

We then assayed the expression level of ZEB1-AS1 in a panel of 114 paraffin embedded prostate cancer specimens. ZEB1-AS1 is associated with later clinical stage (*P* = 0.023) and perineural invasion (*P* = 0.041) in prostate cancer (Table 1). However, there is no significant correlation between ZEB1-AS1 expression and Gleason Score or serum PSA (prostate specific antigen) level.

**Table 1:** Correlation between ZEB1-AS1 and clinicopathological characteristics of prostate cancer

Characteristics	n	ZEB1-AS1 expression		*P* value
		low	high	
Total cases	114	57	57	
Age				
≤ 60	38	16	22	0.221
> 60	76	37	39	
Serum PSA				
≤ 10	31	16	15	0.418
> 10	83	37	46	
TNM stage				
I/II	27	11	16	0.023
III/IV	87	33	54	
Perineural invasion				
Yes	79	30	49	0.041
No	35	14	21	
Gleason score				
≤ 6	23	13	10	0.529
> 6	91	42	49	

### ZEB1-AS1 activates the expression of ZEB1

**Fig. 2:**
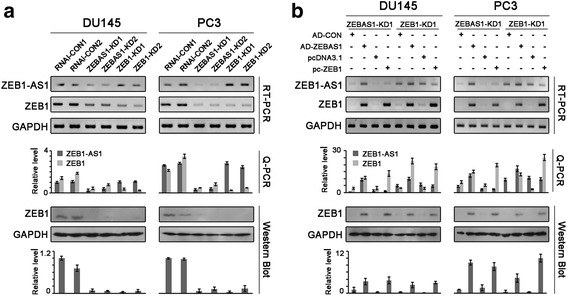
ZEB1-AS1 promoted the expression of ZEB1. **a**, effects of ZEB1-AS1 knockdown on ZEB1. ZEB1-AS1 knockdown in DU145 and PC3 prostate cancer cells (ZEBAS1-KD1 and ZEBAS1-KD2, RNAi-CON1 and RNAi-CON2 as negative control) significantly decreased ZEB1-AS1 mRNA level as well as ZEB1 mRNA and protein levels. ZEB1 knockdown (ZEB1-KD1, ZEB1-KD2) significantly decreased the level of ZEB1 but not ZEB1-AS1. **b**, effects of ZEB1-AS1 re-expression on ZEB1. Adenovirus-mediated ZEB1-AS1 re-expression (AD-ZEBAS1, with AD-CON as negative control) in ZEB1-AS1 knockdown cells significantly recovered ZEB1-AS1 mRNA level as well as ZEB1 mRNA and protein levels. The ZEB1 expression plasmid pc-ZEB1 (pcDNA3.1 as negative control) significantly increased the level of ZEB1 but not ZEB1-AS1 in both ZEB1-AS1 knockdown and ZEB1 knockdown cells

We postulated that the antisense transcript ZEB1-AS1 might regulate the expression of ZEB1 in prostate cancer. RNA interference was used in both DU145 and PC3 cells so as to see the effects of ZEB1-AS1 on ZEB1. ZEB1-AS1 was significantly reduced in ZEB1-AS1 knockdown prostate cancer cells (ZEBAS1-KD1, ZEBAS1-KD2), with concomitant downregulation of ZEB1 mRNA and protein levels. As control, ZEB1 knockdown (ZEB1-KD1, ZEB1-KD2) decreased the level of ZEB1 but not ZEB1-AS1 (Fig. 2a). Furthermore, adenovirus-mediated ZEB1-AS1 re-expression rescued the expressions of ZEB1 in ZEBAS1-KD1 cells, whereas the plasmid pc-ZEB1 overexpressing ZEB1 reversed the level of ZEB1 but not ZEB1-AS1 in both ZEBAS1-KD1 and ZEB1-KD1 cells (Fig. 2b).

### ZEB1-AS1 binds to the H3K4 methyltransferase MLL1 and promotes H3K4me3 histone modification in ZEB1 promoter

**Fig. 3:**
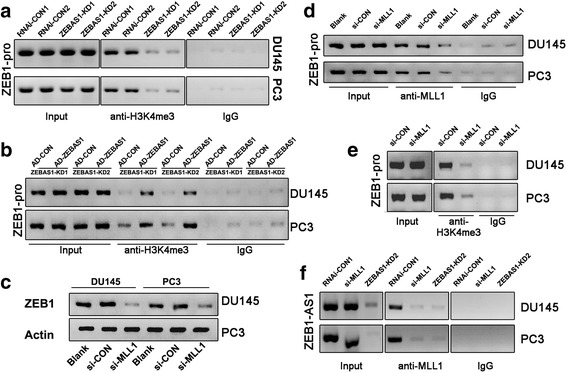
ZEB1-AS1 bound to and recruited MLL1 to maintain H3K4me3 modification in ZEB1 promoter region. A and B, ChIP showed H3K4me3 modification in ZEB1 promoter which was mediated by ZEB1-AS1. PCR using chromatin pulled down by H3K4me3 antibody as template yielded the ZEB1-pro fragment (−1 to −103) of the ZEB1 promoter. ZEB1-AS1 knockdown (ZEBAS1-KD1 and ZEBAS1-KD2) in both DU145 and PC3 cells similarly induced decreased ZEB1 promoter fragment amplified from the precipitates (**a**). In addition, adenovirus-mediated ZEB1-AS1 re-expression (AD-ZEBAS1) rescued this effect (**b**). **c** and **d**, MLL1 mediated the transcription of ZEB1 by combing with its promoter. RT-PCR showed that MLL1 interfering (si-MLL1) inhibited ZEB1 mRNA level in both DU145 and PC3 (**c**). The corresponding ChIP using chromatin pulled down by MLL1 antibody as template showed similar ZEB1-pro attenuation (**d**) as in **a**. **e**, ChIP showed that H3K4me3 modification in ZEB1 promoter region was mediated by MLL1. MLL1 interfering (si-MLL1) in both DU145 and PC3 cells similarly induced decreased ZEB1 promoter fragment amplified from the precipitates. **f**, RIP showed combination of ZEB1-AS1 and MLL1. RT-PCR using RNAs pulled down by MLL1 antibody yielded ZEB1-AS1 fragment. Significant decrease of ZEB1-AS1 level was detected in MLL1 interfering cells (si-MLL1, ZEBAS1-KD2 as negative control)

Preliminary ChIP assays showed H3K4me3 but not H3K9ac or H3K7ac enrichment in ZEB1 promoter region (data not shown). The following ChIP assays using anti-H3K4me3 antibody showed decreased ZEB1 promoter fragment amplification from the precipitates in ZEB1-AS1 knockdown groups (Fig. 3a) and adenovirus-mediated ZEB1-AS1 re-expression rescued this effect (Fig. 3b), which indicated that ZEB1-AS1 was a mediator in the H3K4me3 methylating process of ZEB1 promoter region. SiRNA-mediated inhibition of MLL1, a major methyltransferase responsible for H3K4 trimethylation, suppressed mRNA level of ZEB1 (Fig. 3c). The corresponding ChIP assays with MLL1 antibody showed similar ZEB1-pro attenuation (Fig. 3d) upon MLL1 interference. Those findings illustrated that MLL1 mediated the transcription of ZEB1 by combing with its promoter. We also confirmed that MLL1 mediated the combination of H3K4me3 and ZEB1 promoter by interfering MLL1 (Fig. 3e). RNA-IP assay (RIP) further demonstrated that ZEB1-AS1 RNA could be pulled down by MLL1 antibody in both DU145 and PC3 cells (RNAi-CON1, with ZEBAS1-KD2 and si-MLL1 as negative control), which indicated the physical binding of ZEB1-AS1 with MLL1 (Fig. 3f).

### MiR200c negatively regulates BMI1 in prostate cancer

**Fig. 4:**
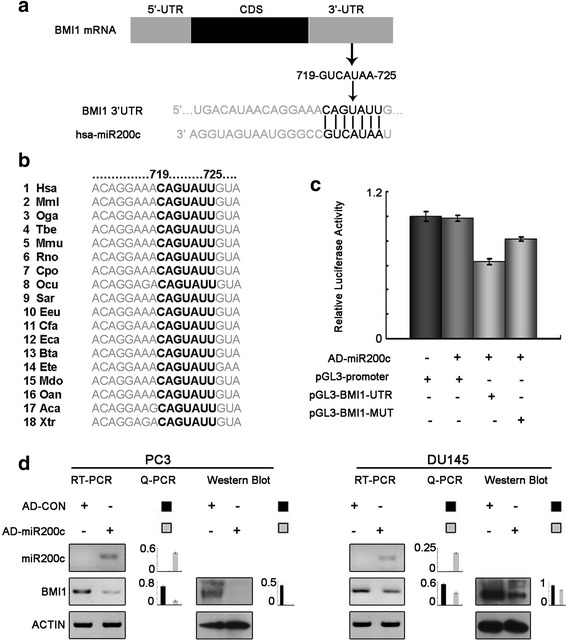
MiR200c negatively regulated BMI1 by targeting corresponding 3′-UTR. **a** and **b**, bioinformatic analysis. The 719–725 nt of the BMI1 3′-UTR was identified as a potential seed sequence for miR200c, and this sequence was conserved across species. **c**, dual reporter gene assays. With artificial expression of miR200c mediated by AD-miR200c, the reporter gene activity was significantly decreased when the seed sequence was present in the construct (pGL3-BMI1-UTR), whereas mutation of the seed sequence (pGL3-BMI1-MUT) significantly restored reporter gene activity. Expression of miR200c had no effect on the blank plasmid pGL3-promoter. **d**, miR200c negatively regulated BMI1. Artificial overexpression of miR200c by AD-miR200c resulted in significant decrease of BMI1 mRNA and protein levels compared with AD-CON

The 1948 nt 3′-UTR of the BMI1 mRNA (full-length 3435 nt, coding sequence 507–1487 nt) was analyzed by using Target Scan 6.2 (http://www.targetscan.org/), which identified miR200c as its potential regulatory microRNA. The 719–725 nt of BMI1 3′-UTR was a potential seed sequence (Fig. 4a) that was highly conserved across species (Fig. 4b). Sequence analysis showed no mutation or deletion of the 3′-UTR in DU145 and PC3 cells. Luciferase reporter gene constructs were prepared in which the potential seed sequence of BMI1 3′-UTR were cloned into luciferase reporter constructs (pGL3–BMI1-UTR), together with constructs in which the seed sequence was mutated (pGL3–BMI1-MUT). With adenovirus-mediated overexpression of miR200c, dual reporter assays showed significant downregulation of luciferase reporter gene activity by 37.2% (*P* < 0.05) in the pGL3–BMI1-UTR construct (pGL3–promoter as negative control). Moreover, mutation of the seed sequences (pGL3–BMI1-MUT) restored the luciferase gene activity by 18.8% (*P* < 0.05) (Fig. 4c). Overexpression of miR200c also induced downregulation of BMI1 mRNA and protein levels (Fig. 4d).

### ZEB1-AS1 inhibits miR200c expression and upregulates BMI1 level

**Fig. 5:**
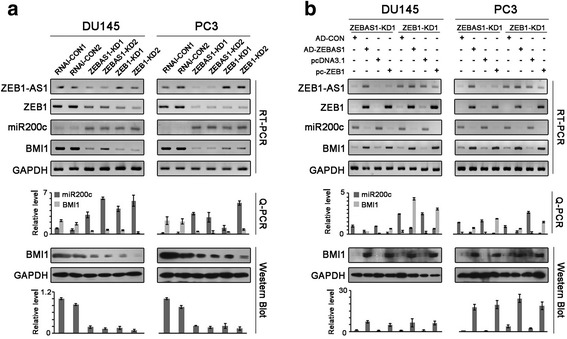
ZEB1-AS1 inhibited miR200c and promoted BMI1 expression. **a**, ZEB1-AS1 knockdown induced increased miR200c level and decreased BMI1 level. ZEB1-AS1 knockdown in DU145 and PC3 prostate cancer cells (ZEBAS1-KD1 and ZEBAS1-KD2) induced significant increase of miR200c and decreased BMI1 mRNA and protein levels. ZEB1 knockdown (ZEB1-KD1, ZEB1-KD2) produced similar effects. **b**, miR200c and BMI1 levels were rebuilded with ZEB1-AS1 re-expression in ZEB1-AS1 knockdown cells. Adenovirus-mediated ZEB1-AS1 re-expression in both ZEB1-AS1 knockdown and ZEB1 knockdown cells comparably induced significant decrease of miR200c level and increased BMI1 mRNA and protein levels. The plasmid pc-ZEB1 overexpressing ZEB1 (pcDNA3.1 as negative control) had the same effects

As is well known, ZEB1 transcriptionally inhibits the expression of miR200c, and in the previous studies, we confirmed the negative regulation effect of miR200c on BMI1. Given the facts above, we furtherly inspected the influences of ZEB1-AS1 on miR200c and BMI1 and as expected, both ZEB1-AS1 and ZEB1 silencing induced miR200c up-regulation and BMI1 inhibition (Fig. 5a). Artificial re-expression of either ZEB1-AS1 or ZEB1 could rescue the effects of ZEB1-AS1 on miR200c and BMI1 (Fig. 5b).

### ZEB1-AS1 knockdown induces proliferation inhibition and migration suppression in prostate cancer

**Fig. 6:**
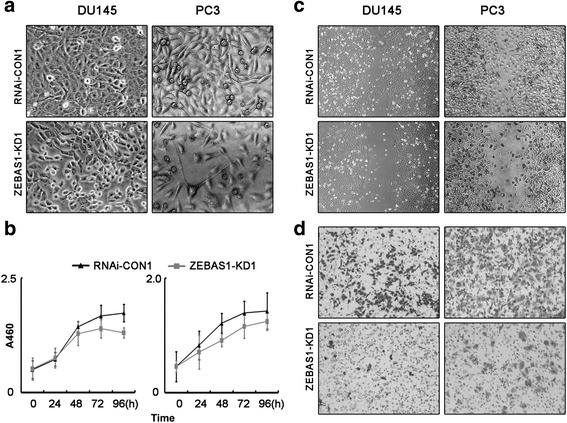
ZEB1-AS1 knockdown induced proliferation inhibition and migration suppression of prostate cancer cells. **a** and **b**, ZEB1-AS1 knockdown induced cell proliferation inhibition. Prostate cancer cells were seeded at the same density in 6-wells plates and photographed after 48 h. The density of ZEB1-AS1 knockdown cells (ZEBAS1-KD1) was obviously lower than those of control (**a**). CCK8 assays (**b**) showed that the growth of both DU145 and PC3 was significantly inhibited concomitant with ZEB1-AS1 knockdown (ZEBAS1-KD1). **c** and D, ZEB1-AS1 knockdown induced cell migration inhibition. Wound healing assays demonstrated that ZEB1-AS1 knockdown induced significant migration inhibition comparing with RNAi-CON1 groups (**c**). For transwell invasion assays, most of the DU145 and PC3 cells invaded from the top chambers to the bottom chambers in RNAi-CON1 groups, but not in the ZEBAS1-KD1 groups (**d**)

Figure 6a showed that the densities of ZEB1-AS1 knockdown cells (ZEBAS1-KD1 typically) were obviously lower than those of control. The growth curves determined by CCK-8 assays showed that ZEB1-AS1 knockdown induced significant cell proliferation inhibition of both DU145 and PC3 (Fig. 6b). Wound healing assay and transwell assay showed that ZEB1-AS1 knockdown prostate cancer cells displayed prominent migration inhibition (Fig. 6c, d). All these data proved the pro-proliferation and pro-migration functions of ZEB1-AS1 in prostate cancer.

## Discussion

The transcriptional factor Zinc finger E-box-binding protein 1 (ZEB1) is overexpressed in various tumors such as endometrial carcinoma, bladder cancer, colorectal cancer, pancreatic cancer and prostate cancer [1–5]. ZEB1, as an epithelial mesenchymal transition (EMT) regulator, together with the EMT associated molecules such as SNAIL, SLUG and TWIST, participates in multiple biological processes of malignancy such as invasion and metastasis [20, 21]. Also, ZEB1 maintains CSC characteristic through regulating the stem cell associated factors such as Sox2, klf4 and Bmi1, which promoting the tumorigenic ability [5, 9]. Besides, ZEB1 also closely related with hypoxia, tumorigenesis and chemosensitivity of tumors [6, 22–25]. In prostate cancer, ZEB1 is correlated with higher Gleason Score and promotes docetaxel-resistance and EMT which is closely related with tumor invasion and migration [4, 26–28]. ZEB1 expression increased according to the different steps of PCa progression and ZEB1 expression in metastases predicted decreased survival of prostate cancer [29].

LncRNAs may originate from different regions of the genome, including the antisense strand or the introns of protein-coding genes, the promoters or untranslated regions of protein-coding genes, or even as independent transcripts within and outside of protein-coding genes [30]. ZEB1-AS1 is an antisense lncRNA derived from the promoter region of ZEB1. In NCBI database, the full length of ZEB1-AS1 was 2568 nt, including two exons (exon1, 200 nt; exon2, 2013 nt) and one intron (355 nt) between the exons. While in this study, the full length of ZEB1-AS1 was 2535 nt, including two exons (exon1, 167 nt; exon2, 2013 nt) and one intron (355 nt) between the exons. The difference between the studies focuses on the initial 33 bp of ZEB1-AS1 missing in our study. In addition, Li et al. reported the length of ZEB1-AS1 was 2449 bp, lacking more in-depth data [16].The discrepancies above might be explained by different cell lines used in different studies or different splicing variants of ZEB1-AS1.

ZEB1-AS1 upregulates ZEB1 expression and is reported to be correlated with progression and poor prognosis of malignancies such as hepatocellular carcinoma, glioma, osteosarcoma and esophageal squamous cell carcinoma [16, 31–33]. Although Liu et al. also claimed that ZEB1 reciprocally inhibited ZEB1-AS1 expression [34], we did not reach the same result. So now the question is, how ZEB1-AS1 upregulates ZEB1?

**Fig. 7:**
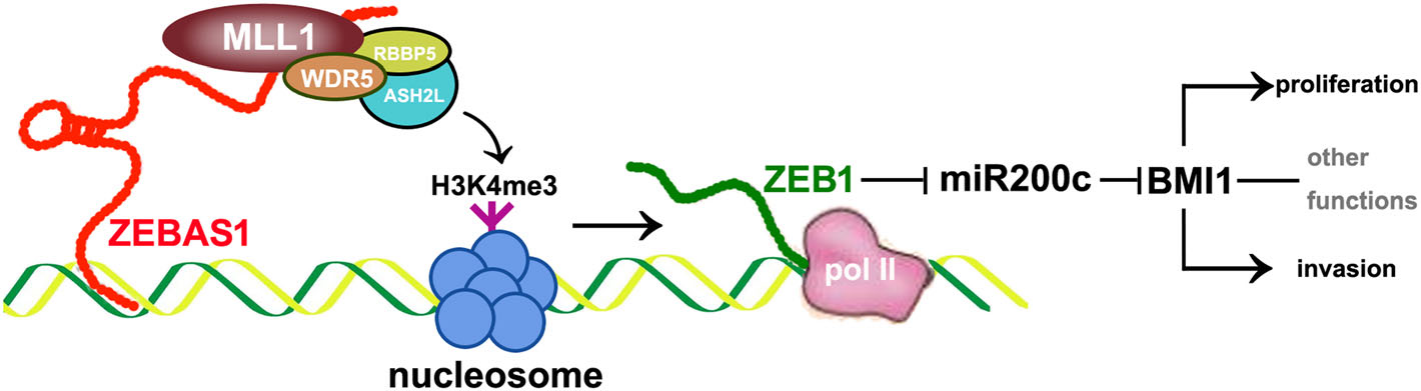
Schematic representation summarizing data from the present study. ZEB1-AS1 was transcribed from the promoter region of ZEB1 in the antisense strand with respect to ZEB1. ZEB1-AS1 recruited the H3K4 methyltransferase MLL1 to ZEB1 promoter and induced H3K4me3 modification in that region. The H3K4me3 modification changed the chromatin status from an inactive state to an active state so as to facilitate ZEB1 transcription. ZEB1 participated in the regulation of malignancy behaviors such as invasion, proliferation and other functions by regulating downstream molecules such as miR200c and BMI1

LncRNAs participate in various pathophysiological processes such as gene silencing, histone modifying, enhancer activity organizing and genomic reprogramming. Notably, epigenetic modifications such as histone methylation and acetylation are frequently observed in regulatory processes mediated by lncRNAs. For instance, the lncRNA from the antisense of the gene p21 induced H3K27 methylation in the promoter region of the sense strand, which induced p21 transcription inhibition [15]. A lncRNA transcribed from the 5′ promoter region of CCND1 recruits TLS (for translocated in liposarcoma) to the CCND1 promoter by inhibiting histone acetyltransferase activities and caused CCND1 transcription inhibition [35]. Liu et al. reported that ZEB1-AS1 directly binds and recruits the histone acetyltransferase p300 to the ZEB1 promoter region and activates ZEB1 transcription [33]. The glaring issue here is trimethylated lysine 4 of histone H3 (H3K4me3). H3K4me3 marks the transcription start sites of actively transcribed genes and plays a major role in facilitating transcription initiation [36]. The methyltransferases specifically directing at H3K4 are MLL family proteins (MLL1/2/3/4,SET1A/B). Work in mammalian H3K4 methylation has primarily focused on MLL1, the function of which depends on the participation of the helper proteins WDR5, RBBP5 and ASH2L. With a series of immunoprecipitation assays, we found that ZEB1-AS1 mediated H3K4 trimethylation in ZEB1 promoter region by binding to and recruiting MLL1, although the specific interacting mode between ZEB1-AS1 and MLL1 needs further investigation. H3K4me3 transition in ZEB1 promoter will change the chromatin status there from a closed and inactive state to an open and active state and facilitate ZEB1 transcription (Fig. 7).

In addition, ZEB1 binds to at least two highly conserved sites in the putative promoter of miR200c and inhibited its expression [37]. Conversely, miR200c directly suppressed ZEB1 expression by binding with the seed sequences in ZEB1 3′-UTR [38, 39]. They are reciprocally linked in a feedback loop, each strictly controlling the expression of the other [28]. This feedback loop is deregulated in several human cancers including those of breast, pancreatic, colon and also prostate. In this study, ZEB1-AS1 indirectly inhibited the tumor suppressor miR200c, and furtherly promoted the expression of BMI1, the degradation of which was mediated by directly binding of miR200c [5, 13]. It is worth mentioning that overexpression of BMI1 correlates with therapy failure in many tumors including those of breast, lung, liver and colon and targeting BMI1 by gene therapy abolishes chemoresistance in tumor cells. Also, BMI1 promotes cell survival and attenuated chemosensitivity to docetaxelin prostate cancer [40]. The ZEB1-miR200c-BMI regulation pathway may play an important role in ZEB1-AS1-mediated prostate cancer cell proliferation and migration.

## Conclusions

In summary, lncRNA ZEB1-AS1 binds to and recruits MLL1 to the promoter region of ZEB1, inducing H3K4me3 therein and activates ZEB1 transcription. The ZEB1-AS1-ZEB1-miR200c-BMI1 pathway may be an important part of the dysfunctional regulatory network in prostate cancer.

## Abbreviations

CHIP: Chromatin immunoprecipitation
lncRNA: Long non-coding RNA
RACE: Rapid amplification of cDNA ends
RIP: RNA Immunoprecipitation
ZEB1-KD: ZEB1 knockdown
ZEBAS1-KD: ZEB1-AS1 knockdown
